# Cervical percutaneous interferential current stimulation improves citric acid cough tests in patients with Parkinson’s disease on medication

**DOI:** 10.1038/s41598-024-62460-x

**Published:** 2024-05-18

**Authors:** Masahiro Nakamori, Megumi Toko, Hidetada Yamada, Yuki Hayashi, Kai Ushio, Kohei Yoshikawa, Aya Hiraoka, Mineka Yoshikawa, Toshikazu Nagasaki, Yoshitaka Shimizu, Yukio Mikami, Hirofumi Maruyama

**Affiliations:** 1 Department of Clinical Neuroscience and Therapeutics, Hiroshima University Graduate School of Biomedical and Health Sciences, Hiroshima, Japan; 2 Department of Rehabilitation Medicine, Hiroshima University Hospital, Hiroshima, Japan; 3 Department of Advanced Prosthodontics, Hiroshima University Graduate School of Biomedical and Health Sciences, Hiroshima, Japan; 4 Department of Oral and Maxillofacial Radiology, Hiroshima University Graduate School of Biomedical and Health Sciences, Hiroshima, Japan; 5 Department of Dental Anesthesiology, Hiroshima University Graduate School of Biomedical and Health Sciences, Hiroshima, Japan

**Keywords:** Health care, Neurology

## Abstract

Aspiration pneumonia is the leading cause of death in patients with Parkinson’s disease. The incidence of silent aspiration is high in such patients owing to decreased pharyngeal and laryngeal sensation; thus, interventions for this condition may help prevent pneumonia. In this single-arm, open-label study, we used a cervical percutaneous interferential current stimulation device to activate pharyngeal and laryngeal sensory nerves. We evaluated its effectiveness in patients with Hoehn–Yahr stages 2–4 Parkinson’s disease. The primary endpoint was the proportion of patients with a normal cough reflex after consuming 1% citric acid at the end of the intervention compared with baseline measurements. In total, 25 patients received neck percutaneous interferential current stimulation for 20 min twice weekly for 8 weeks. Afterward, the proportion of patients with a normal cough reflex after 1% citric acid consumption increased significantly (*p* = 0.001), whereas other indicators, such as tongue pressure, peak expiratory flow, and penetration or aspiration during videofluoroscopic examination, remained unchanged. A longer duration of illness, higher Unified Parkinson’s Disease Rating Scale total scores, and higher levodopa equivalent daily doses were significantly associated with improved cough test outcomes. Hence, cervical percutaneous interferential current stimulation significantly improved cough reflexes and may improve silent aspiration.

**Trial Registration:** Japan Registry of Clinical Trials, jRCTs062220013, first registered 09/05/2022.

## Introduction

Neurological disorders often induce dysphagia, elevating the risk of aspiration pneumonia, affecting patients’ prognosis and outcomes, and consequently increasing medical and caregiving costs. Among these neurological disorders, Parkinson’s disease (PD), often referred to as “Parkinson’s pandemic,” is one of the most common diseases with a rapidly rising global incidence^1^. Aspiration pneumonia is a leading cause of death in patients with PD. Therefore, evaluating and developing appropriate and effective interventions for preventing pneumonia are crucial. In particular, patients with PD have a high incidence of silent aspiration owing to decreased pharyngeal and laryngeal sensation^2^, necessitating interventions for the prevention of pneumonia. Bedside screening methods, such as the citric acid cough test, are frequently used for evaluating silent aspiration, with impaired cough reflexes increasing the risk of aspiration pneumonia^3^.

Treating the underlying disease is the top priority in treating swallowing disorders associated with PD. The treatment of PD mainly involves pharmacotherapy with anti-parkinsonian drugs. However, a combination of interventions is essential to improve and maintain function. Swallowing rehabilitation mainly focuses on strengthening swallowing-related muscle groups, and training is conducted to strengthen the tongue and suprahyoid muscles^4^. Conversely, effective therapy for decline in sensory function, including silent aspiration, has been performed using techniques such as ice massage^5^. However, evidence-based approaches are limited, and effective methods have not been developed because of the nature of sensory nerves.

Recently, several instruments have been designed to stimulate and modulate neurological functions. One such innovation involves percutaneous electrical neck stimulation, which can enhance neuromuscular function. Various approaches utilizing pulsed current stimulation have proven effective in inducing muscle contractions, showing promise in treating diverse dysphagia conditions and gaining widespread application in clinical settings^6^. However, a notable drawback of this method is the pain and invasiveness associated with muscle contractions. An alternative approach, employing interferential current sensory stimulation, has been developed. Interferential current stimulation is believed to create undulations locally by superimposing two slightly different mid-frequency electrical stimuli, resulting in interference waves that reach deep tissues^7^. In a double-blind, randomized, comparative trial in humans, the percutaneous interferential current stimulation group showed a significant increase in the number of swallows during 5 min of 3 mL/min water infusion into the pharynx compared to the control group, reducing pharyngeal latency and indicating that interferential current facilitates the swallowing reflex^8^. Contrarily, in experiments with anesthetized guinea pigs, the cough and swallowing reflexes triggered by laryngeal citric acid or current stimulations were eliminated by transecting the laryngeal and tracheal afferent nerves. Additionally, these reflexes scan be induced by stimulating transected nerve endings or the tracheal mucosa^9^. Hence, this animal experiment showed that current stimulation activates peripheral sensory nerves in the pharynx and larynx, thereby inducing cough and swallowing reflexes through the same neural stimulation as citric acid challenges. Some studies suggest that these electrical stimulation devices can enhance swallowing without necessitating muscle contractions^10,11^. Therefore, they can stimulate sensory nerves in the deeper layers of the pharynx and larynx without causing discomfort, making interferential current stimulation a promising approach for alleviating dysphagia^8^. In addition, effects such as enhanced saliva production and improved nutrition in patients with dysphagia have been reported with interferential current stimulation^12,13^. The facilitation of the cough reflex through the use of such a device is crucial from the perspectives of airway clearance, protection against aspiration, and prevention of aspiration pneumonia.

However, previous reports have not focused on specific diseases or conditions, and have instead targeted a broad range of patients with swallowing disorders. The effects of the device may vary depending on the specific disease or condition. In this study, we investigated the effects of interferential current stimulation in patients with PD during outpatient visits and evaluated their swallowing function by use of a cough test.

## Results

In this study, 27 participants were initially enrolled; however, two individuals withdrew their consent for personal reasons within 4 weeks of participating in the study. Consequently, interventions and evaluations were conducted on 25 participants without deviation. Adverse events related to the intervention, such as worsened cervical skin or neurological symptoms due to stimulation, did not occur.

**Figure 1:**
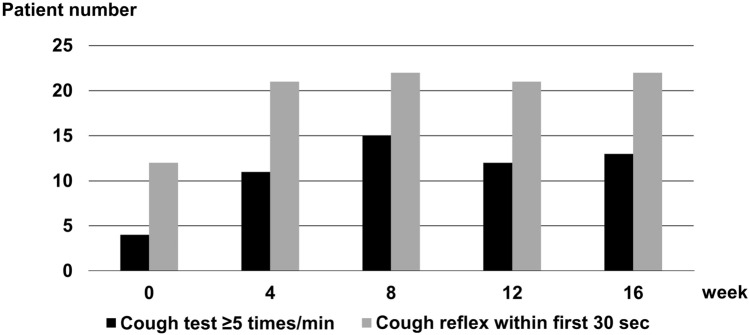
Transition of normal individuals in the cough and simplified cough tests.

Table 1 presents patient demographics, cough test results, and baseline data for swallowing-related indicators (pre-intervention), as well as details on the transition of indicators at baseline, the end of the intervention (8 weeks from the initiation), and 8 weeks after the last intervention (16 weeks from the initiation). Anti-parkinsonian medication demonstrated minimal changes throughout the study period. The normal proportions in the cough and simplified cough tests significantly increased over the 8-week intervention (*p* = 0.001 and 0.002, respectively). Figure 1 illustrates the transition of individuals with normal results for the cough and simplified cough tests, which were evaluated every 4 weeks. The χ^2^ test revealed significant improvements in the cough and simplified cough tests at 4, 8, 12, and 16 weeks compared with baseline (0 weeks) (*p* < 0.05). Most patients had an Functional Oral Intake Scale (FOIS) score of 7, except for one with a score of 6. The patient with a FOIS score of 6 had an Eating Assessment Tool-10 (EAT-10) score > 3 (indicative of abnormal swallowing). After the 8-week intervention, a significant increase in the number of individuals classified as normal was observed (EAT-10 score ˂ 3; (*p* = 0.002). Moreover, 8 weeks after the intervention (16 weeks from initiation), the proportions of individuals classified as normal in the cough test, simplified cough test, and EAT-10 remained significantly higher than those at baseline (*p* = 0.007, 0.002, and 0.007, respectively). In contrast, other swallowing-related indicators, such as tongue pressure, peak expiratory flow, and videofluoroscopic examinations (VF) penetration or aspiration, remained stable throughout the study. No cases of pneumonia were observed throughout the study period.

**Table 1:** Patient characteristics and transition of study indicators at baseline, 8 weeks, and 16 weeks from initiation of intervention.

Indicators	0 week (pre-intervention)	8 weeks (post-intervention)	*p*-value	16 weeks	*p*-value
Age (years)	72.0 ± 5.9				
Sex (female), n (%)	9 (36.0)				
Duration (years)	6 (1, 20)				
Alcohol consumption, n (%)	2 (8.0)				
Current smoking, n (%)	3 (12.0)				
Body mass index (kg/m^2^)	21.2 ± 2.8	21.3 ± 2.8	0.899	21.0 ± 2.5	0.782
Hoehn–Yahr stage	3 (2, 4)	3 (2, 4)	0.788	3 (2, 4)	0.788
UPDRS score (total)	37 (19, 76)	42 (14, 78)	0.853	44 (15, 80)	0.554
UPDRS score (part 3)	23 (10, 50)	25 (10, 51)	0.930	28 (11, 51)	0.669
Levodopa (mg)	360 ± 203	360 ± 203	1.000	360 ± 203	1.000
LEDD (mg)	583 ± 395	583 ± 395	1.000	589 ± 392	0.957
Maximum handgrip strength (kg)	24.8 ± 5.6	25.5 ± 5.4	0.665	26.7 ± 6.4	0.276
Calf circumference (cm)	33.8 ± 3.3	33.8 ± 3.5	1.000	34.0 ± 3.2	0.830
FOIS	7 (6, 7)	7 (6, 7)	1.000	7 (6, 7)	1.000
EAT-10 < 3, n (%)	12 (48.0)	22 (88.0)	0.002*	21 (84.0)	0.007*
Tongue pressure (kPa)	30.6 ± 8.5	33.6 ± 8.0	0.199	34.6 ± 8.4	0.101
Peak expiratory flow (L/min)	196.9 ± 76.7	233.9 ± 93.2	0.132	226.3 ± 88.6	0.216
Cough test ≥ 5 times/min, n (%)	4 (16.0)	15 (60.0)	0.001*	13 (52.0)	0.007*
Cough reflex within first 30 s, n (%)	12 (48.0)	22 (88.0)	0.002*	22 (88.0)	0.002*
Penetration or aspiration on VF	10 (40.0)	9 (36.0)	0.771	11 (44.0)	0.775
Hemoglobin (g/dL)	13.2 ± 1.2	13.0 ± 1.2	0.469	13.2 ± 1.3	0.982
White blood cell (μL)	6092 ± 1629	5718 ± 1687	0.430	5592 ± 1313	0.238
C-reactive protein (mg/dL)	0.10 ± 0.15	0.11 ± 0.32	0.784	0.06 ± 0.10	0.319
Albumin (g/dL)	4.2 ± 0.3	4.0 ± 0.3	0.139	4.1 ± 0.2	0.339
Total cholesterol (mg/dL)	204.4 ± 31.9	200.2 ± 30.5	0.637	204.6 ± 32.2	0.986

Data are expressed as mean ± standard deviation or median (minimum, maximum) for continuous variables and frequencies and percentages for discrete variables. Univariate analyses were performed to compare the baseline (0 weeks). *p < 0.05 is considered statistically significant.

*UPDRS* Unified Parkinson’s Disease Rating Scale, *LEDD* levodopa equivalent daily dose, *FOIS* Functional Oral Intake Scale, *EAT-10* Eating Assessment Tool-10, *VF* videofluoroscopic examination.

Chi-square test revealed significant improvements in the cough and simplified cough tests at 4, 8, 12, and 16 weeks compared to baseline (0 weeks) (*p* < 0.05).

Overall, 21 participants had abnormal cough test results at baseline, and 12 showed improvements within the normal range by the end of the 8-week intervention (Table 2). Therefore, a univariate analysis was conducted using the baseline data to examine factors associated with improvement between the 12 individuals who showed improvement and the nine who did not. Longer illness duration, higher Unified PD Rating Scale total scores (UPDRS), and higher levodopa equivalent daily doses (LEDD) were significantly associated with improved cough test outcomes (*p* = 0.049, 0.049, and 0.046, respectively). The correlation coefficients between the duration of illness and UPDRS total score, UPDRS total score and LEDD, and LEDD and the duration of illness were 0.551 (*p* = 0.010), 0.668 (*p* = 0.001), and 0.547 (*p* = 0.010), respectively. These three factors exhibited strong inter-correlations, making multivariate analysis challenging. In contrast, four participants had normal cough test results at baseline, but one individual transitioned to an abnormal state at the end of the 8-week intervention. The frequency of cough reflexes per minute decreased from five to three times. However, the simplified cough test remained normal, and this patient had an LEDD of 300 mg.

**Table 2:** Comparison between the improvement and non-improvement groups for the cough test.

Indicators	Improvement (n = 12)	Non-improvement (n = 9)	*p*-value
Age (years)	71.3 ± 6.5	71.9 ± 5.9	0.819
Sex (female), n (%)	4 (33.3)	3 (33.3)	1.000
Duration (years)	10 (4, 20)	5 (1, 13)	0.049*
Body mass index (kg/m^2^)	21.4 ± 2.9	21.8 ± 2.8	0.783
Alcohol consumption, n (%)	0 (0)	1 (11.1)	0.237
Current smoking, n (%)	2 (16.7)	1 (11.1)	0.719
Hoehn–Yahr stage	3 (2, 4)	3 (2, 3)	0.298
UPDRS score (total)	50 (21, 76)	28 (19, 54)	0.049*
UPDRS score (part 3)	30 (14, 50)	18 (10, 33)	0.154
Levodopa (mg)	433 ± 214	306 ± 142	0.138
LEDD (mg)	765 ± 444	414 ± 243	0.046*
Maximum handgrip strength (kg)	25.4 ± 4.3	25.3 ± 7.7	0.975
Calf circumference (cm)	34.0 ± 3.5	34.4 ± 3.1	0.765
FOIS	7 (6, 7)	7 (7, 7)	1.000
EAT-10 < 3, n (%)	7 (58.3)	4 (44.4)	0.528
Tongue pressure (kPa)	31.6 ± 8.9	28.2 ± 6.2	0.340
Peak expiratory flow (L/min)	213.3 ± 73.3	211.6 ± 74.8	0.959
Penetration or aspiration on VF, n (%)	5 (41.7)	4 (44.4)	0.899
Hemoglobin (g/dL)	13.5 ± 1.3	12.9 ± 1.2	0.299
White blood cell (μL)	6309 ± 1680	5834 ± 1886	0.550
C-reactive protein (mg/dL)	0.14 ± 0.19	0.07 ± 0.10	0.325
Albumin (g/dL)	4.1 ± 0.2	4.3 ± 0.3	0.148
Total cholesterol (mg/dL)	195.7 ± 26.8	211.4 ± 38.0	0.306

Data are expressed as mean ± standard deviation or median (minimum, maximum) for continuous variables and frequencies and percentages for discrete variables. Univariate analyses were performed to compare the improvement group with the non-improvement group. *p < 0.05 is considered statistically significant.

*UPDRS* Unified Parkinson’s Disease Rating Scale, *LEDD* Levodopa equivalent daily dose, *FOIS* Functional Oral Intake Scale, *EAT-10* Eating Assessment Tool-10, *VF* videofluoroscopic examination.

Finally, we investigated the presence of coughing or throat clearing, and the results of the cough test when penetration or aspiration was observed on VF. At baseline, among the 10 cases showing penetration or aspiration on VF, two exhibited coughing or throat clearing, with one patient each having normal and abnormal cough reflexes. Contrarily, among the eight cases that did not exhibit coughing or throat clearing, all had abnormal cough reflexes. At the end of the 8-week intervention, among the nine cases showing penetration or aspiration on VF, six exhibited coughing or throat clearing, and all had normal cough reflexes. On the other hand, among the three cases that did not exhibit coughing or throat clearing, two had abnormal cough reflexes. At baseline, the chi-squared test result was *p* = 0.035, and at the end of the 8-week intervention, it was *p* = 0.023.

## Discussion

This study used a cervical nerve electrical stimulation device to activate sensory nerves in the pharynx and larynx and evaluated the effectiveness of cervical percutaneous interferential current stimulation in enhancing swallowing function in patients with Hoehn–Yahr stages 2–4 PD. To the best of our knowledge, this is the first trial exclusively conducted on patients with PD. PD leads to various swallowing disorders, including abnormal transport from the oral cavity and pharynx^24^, delayed swallowing reflex^25^, and pharyngeal residue^2^. Among these, pharyngeal and laryngeal hypoesthesia are serious and characteristic of PD^2^. Strengthening the swallowing-related muscles, such as the tongue, soft palate, and suprahyoid muscles, is a useful and established method for swallowing rehabilitation. However, effective methods for preventing silent aspiration or improving sensory functions have not been established.

In this study, cervical percutaneous interferential current stimulation significantly increased cough test normalcy, suggesting its potential in improving silent aspiration. Additionally, the presence or absence of coughing or throat clearing during penetration or aspiration on VF was found to be significantly associated with the results of the cough reflex, confirming the correlation between cough test and defense against aspiration. The clinical significance of cough reflex improvement or throat clearing through cervical percutaneous interferential current stimulation, based on swallowing dynamics evaluation via VF, is extremely important. Additionally, this study observed improvements in EAT-10 scores following intervention. The improvement in subjective swallowing status in patients not only prevents aspiration pneumonia but also improves the quality of life related to meals. This includes enhancements in oral intake and nutritional status, suggesting potential ripple effects that could be anticipated. Furthermore, this effect persisted for an additional 8 weeks after the intervention, indicating the possibility of neuromodulation due to stimulation. Future research should observe the long-term sustainability of this effect. Additionally, investigating whether improvements in cough test results contribute to a reduction in pneumonia incidence by observing a large number of cases over an extended period is essential.

In contrast, tongue pressure, peak expiratory flow, and VF measurements of penetration or aspiration did not show significant changes. Cervical percutaneous interferential current stimulation, adopted in this study, focused on sensory nerve stimulation. Tongue pressure and peak expiratory flow were indicators of muscle strength. The equipment used in this study was not intended for exercise or muscle enhancement; therefore, the absence of improvements was not contradictory. The stability in the percentages of aspiration and laryngeal penetration observed via VF can also be attributed to the multifactorial nature of dysphagia in PD, which includes motor function. Therefore, the stimulation used in this study alone could not address the numerous factors. Multiple interventions from various perspectives are required to address dysphagia in PD.

A notable finding was that comparing participants with improved cough test results with those without improvements revealed that those with longer disease duration had higher Unified PD Rating Scale scores and LEDD values, which was unexpected and remarkable. A possible explanation is that levodopa stimulation induces substance P^26,27^, which triggers a cough reflex. The high LEDD values potentially increased substance P levels and may have contributed to the improved cough reflex through cervical electrical stimulation. Measuring substance P levels in blood and saliva could validate this hypothesis in the future.

This study has some limitations. First, this was a single-site, single-group intervention trial. We should consider establishing a non-intervention or sham stimulation group for intergroup comparisons in the future. This study is the first to investigate the effectiveness and safety of cervical interferential current stimulation, successfully achieving its primary objectives. Despite the ethical challenges in the future, intergroup comparison trials should be conducted. Second, in this study, we targeted patients with relatively mild PD who receive outpatient care and were not hospitalized or residing in facilities. Therefore, expanding the results of this study to severe patients may not be appropriate for generalization. The reason for this choice was the ethical consideration of the risks associated with conducting examinations including VF. Another reason was the belief that including a wide range of patients from mild to severe PD would make the interpretation of the intervention effects challenging. Even in mild cases, the frequency of swallowing disorders is high, emphasizing the importance of identifying the risk and intervening at an early stage^2^. Actually, we observed a significant number of patients with reduced cough reflex, and we believe the significance of the intervention was identified. Third, in this study, improvements in cough reflex and EAT-10 were observed through intervention. However, notably, EAT-10 relies on subjective assessment through a questionnaire, raising concerns about its reliability. However, in this study, for evaluation swallowing, we used not only the subjective assessment through the EAT-10 questionnaire but also objective measures such as cough tests, tongue pressure, and the VF, which is the gold standard for swallowing measurement. Moreover, we assessed PD symptoms using parameters such as the duration of illness, medication dosage, Hoehn–Yahr stage, and UPDRS. These objective measures are widely used and accepted in research on PD. Fourth, the intervention intensity was limited. In this study, we targeted relatively stable outpatients with PD who received interventions twice a week. Therefore, the effects may have been insufficient. Nevertheless, the significant improvement in the citric acid cough test scores, an indicator of silent aspiration, was a meaningful result. Therefore, multiple intervention densities for this examination should be assessed in future studies.

In conclusion, cervical percutaneous interferential current stimulation significantly improved cough reflexes, suggesting a potential contribution in mitigating silent aspiration in patients with PD. This might contribute to reducing the risk of pneumonia, improving the quality of life related to meals, and enhancing nutritional status. Future research should incorporate randomization, explore stimulatory conditions, and include physiological and biochemical evidence to further investigate this phenomenon. Furthermore, clinical studies should focus on investigating whether our findings can be generalized to other neurological disorders and geriatric dysphagia along with PD.

## Material and methods

### Ethics approval, registrations, and patient consent

This study was approved by the Hiroshima University Certified Review Board (approval number: CRB6180006) and was conducted in accordance with the guidelines of the national government based on the Helsinki Declaration of 1964. This study has been registered with jRCT (trial registration number: jRCTs062220013, first registered 09/05/2022). The recruitment period for this study was from May 9, 2022 to June 30, 2023. The explanation to participants was conducted using written documents, and written consent was obtained. All participants provide written informed consent before the study.

### Study design and protocol

The study design and protocol have been previously published^14^. This single-arm, open-label study was conducted at Hiroshima University Hospital and adhered to the Standard Protocol Items: Recommendations for Interventional Trials reporting guidelines^15^. We investigated the efficacy and safety of cervical percutaneous interferential current stimulation in patients diagnosed with Hoehn–Yahr stages 2–4 PD based on the Movement Disorder Society criteria^16^. We included patients diagnosed with clinically probable or established PD (Hoehn–Yahr stages 2–4) at registration, based on the Movement Disorder Society criteria and their ability to visit the hospital twice a week and provide written informed consent. The inclusion criteria were as follows: (1) stable levodopa dosage for over 1 month and (2) age between 20 and 85 years. The exclusion criteria were as follows: (1) implantation with pacemakers or implantable defibrillators as the instruction manual states that the procedure is contraindicated in such patients, (2) undergoing treatments with deep brain stimulation, (3) pregnant or attempting to become pregnant, (4) diagnosed with or having a history of head or neck cancer, (5) active pneumonia, and (6) a history of swallowing rehabilitation.

**Figure 2:**
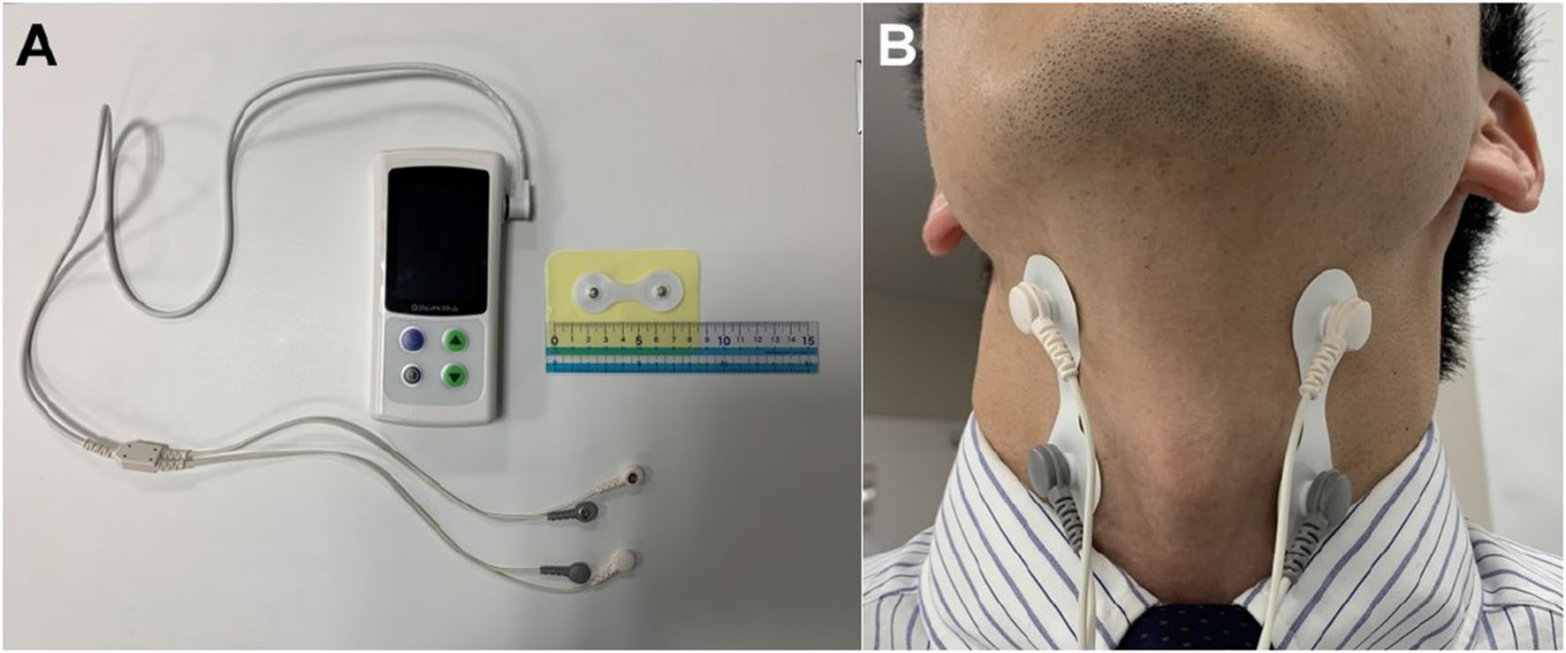
Photos depicting the device and its attachment to the neck.

The enrolled patients underwent cervical percutaneous interferential current stimulation for 20 min twice weekly for 8 weeks utilizing a Gentle Stim® (FoodCare Co., Ltd., Kanagawa, Japan) device. Figure 2 shows the device as well as its attachment to the neck. Pads were attached to the front of the neck from the lower border of the mandibular angle to the anterior margin of the sternocleidomastoid muscle. This device has an output of 2050 Hz from one pair of electrodes and 2000 Hz from another pair, causing interference within the deep part of the neck and generating a low-frequency interference current (50 Hz) corresponding to the difference in frequencies in the affected area. Thus, the interferential current (50 Hz) had a lower stimulation threshold than that of pulse stimulation, resulting in minimal patient sensations. A unified protocol was followed for stimulation in all the patients, and a maximum stimulation current below the perceived electrical stimulation threshold (2.0–2.5 mA) was utilized.

Evaluations were performed every 4 weeks from the beginning of the intervention to 16 weeks after intervention initiation, excluding VF, which were conducted every 8 weeks from the beginning of the intervention to 16 weeks after. The primary endpoint was the proportion of patients exhibiting a normal cough reflex, defined as coughing five times or more in 1 min after a 1% citric acid cough challenge at the end of the intervention (8 weeks from initiation of intervention). The secondary endpoints were: (1) proportion of patients with a normal cough reflex after the 1% citric acid challenge, 8 weeks after the last intervention (16 weeks from the initiation); (2) proportion of patients with a cough reflex after the 1% citric acid challenge within the first 30 s (simplified cough test) after the intervention (8 weeks from initiation of intervention); (3) proportion of patients with a cough reflex after the 1% citric acid challenge within the first 30 s (simplified cough test), 8 weeks after the last intervention (16 weeks from initiation of intervention); (4) proportion of patients with normal swallowing status at the end of the intervention (8 weeks from initiation of intervention) (normal swallowing status was defined as an FOIS score of 7 and an EAT-10 score < 3); (5) proportion of patients with normal swallowing status 8 weeks after the last intervention (16 weeks from initiation of intervention); (6) proportion of aspiration and penetration observed via VF examination at the end of the intervention (8 weeks from initiation of intervention); (7) proportion of patients with aspiration and penetration revealed via VF examination 8 weeks after the last intervention (16 weeks from initiation of intervention); and (8) incidence of the onset of pneumonia during the 16-week period after initiation of intervention. Safety was evaluated based on skin symptoms at region of electrode attachment and exacerbation of neurological symptoms owing to electrical stimulation of the head and neck.

### Cough and simplified cough tests

A cough test was performed while patients inhaled 1% citric acid–physiological saline mist using a portable mesh nebulizer (NE-U22, Omron, Kyoto, Japan). Citric acid was purchased from Kenei Pharmaceutical Co. Ltd. (Osaka, Japan). Patients were verbally instructed to deeply inhale the nebulized citric acid through their mouths. In the original cough test, five or more coughs occurring within 1 min were considered normal^17^. In the simplified cough test, the first cough reflex occurring within 30 s was considered normal^18^.

### VF

VF tests were performed using an X-ray imaging system (Ultimax-I, Canon Medical Systems, Tochigi, Japan) with the patient in a seated position. The test involved delivering 3 mL of water containing 30%/w barium contrast medium (Barytester A240 Powder®, FUSHIMI Pharmaceutical Co. Ltd., Kagawa, Japan) to the floor of the mouth of the patients via a syringe and instructing them to swallow. The X-ray system captured images forward toward the lips, backward to the pharyngeal wall, upward to the nasal cavity, and downward to the upper esophageal sphincter. A side-VF recording at 30 frames per second was obtained and stored on a digital video disc. Two dentists (AH and MY) with specialized experience in evaluating VF recordings, who were blinded to the study, determined the presence or absence of aspiration and laryngeal penetration. The two observers discussed their observations and reached a consensus regarding each observation or measurement.

### Data acquisition

Two neurologists (MN and HY) conducted clinical evaluation and diagnosis. The recorded data included body mass index, grip power, calf circumference, disease duration, alcohol consumption, smoking habits, UPDRS score^19^, medication, FOIS score, EAT-10 score, and blood test results^20^. Tongue pressure and peak expiratory flow were assessed using previously reported methods^21,22^. LEDDs were calculated based on the most recent study^23^. All evaluations were conducted in the ON state.

### Sample size calculation

Calculations were performed to determine the necessary sample size based on preliminary cough tests conducted among elderly patients with neurodegenerative disorders. The initial assessment revealed that 28.6% of patients exhibited a normal cough reflex after undergoing the 1% citric acid challenge. We estimated a sample size of 27 participants, assuming that the proportion of patients with a normal cough reflex would increase to 50% after 8 weeks of therapy. This estimation considered a 0.10 alpha level, 0.80 power, and an anticipated 10% dropout rate.

### Statistical analysis

The safety analysis involved assessment of the occurrence of skin symptom and aggravation of neurological symptoms resulting from electrical stimulations in the cranial and cervical regions. To evaluate the primary and secondary endpoints, the swallowing status of each patient before the initial intervention was compared with their status at the end of the intervention (8 weeks from initiation) or 8 weeks after the intervention (16 weeks from initiation). Furthermore, a statistical comparison was conducted between the cough test improvement and non-improvement groups. Statistical analyses were performed using JMP version 16 (SAS Institute Inc., Cary, NC, USA). The chi-squared (χ^2^), Mann–Whitney U, or unpaired t-test was employed to assess the statistical significance of intergroup differences. The factors presumed to have a strong correlation were examined by calculating the Spearman rank correlation coefficient. Statistical significance was set at *p* < 0.05.

## Data Availability

Data supporting the findings of this study are available from the corresponding author upon request.

## References

[1] Dorsey ER, Sherer T, Okun MS, Bloem BR (2018). The emerging evidence of the Parkinson pandemic. J. Parkinsons Dis..

[2] Bushmann M, Dobmeyer SM, Leeker L, Perlmutter JS (1989). Swallowing abnormalities and their response to treatment in Parkinson’s disease. Neurology.

[3] Nakamori M (2020). Simplified cough test can predict the risk for pneumonia in patients with acute stroke. PLoS One.

[4] Liu J (2022). Effects of chin tuck against resistance exercise on post-stroke dysphagia rehabilitation: A systematic review and meta-analysis. Front. Neurol..

[5] Nakamura T, Fujishima I (2013). Usefulness of ice massage in triggering the swallow reflex. J. Stroke Cerebrovasc. Dis..

[6] Carnaby GD, Harenberg L (2013). What is “usual care” in dysphagia rehabilitation: A survey of USA dysphagia practice patterns. Dysphagia.

[7] Petrofsky J (2008). The effect of the subcutaneous fat on the transfer of current through skin and into muscle. Med. Eng. Phys..

[8] Furuta T, Takemura M, Tsujita J, Oku Y (2012). Interferential electric stimulation applied to the neck increases swallowing frequency. Dysphagia.

[9] Tsujimura T, Udemgba C, Inoue M, Canning BJ (2013). Laryngeal and tracheal afferent nerve stimulation evokes swallowing in anaesthetized guinea pigs. J. Physiol..

[10] Zhang M (2016). Effectiveness of neuromuscular electrical stimulation on patients with dysphagia with medullary infarction. Arch. Phys. Med. Rehabil..

[11] Ortega O, Rofes L, Martin A, Arreola V, López I, Clavé P (2016). A comparative study between two sensory stimulation strategies after two weeks treatment on older patients with oropharyngeal dysphagia. Dysphagia..

[12] Hasegawa Y, Sugahara K, Sano S, Sakuramoto A, Kishimoto H, Oku Y (2016). Enhanced salivary secretion by interferential current stimulation in patients with dry mouth: A pilot study. Oral Surg. Oral Med. Oral Pathol. Oral Radiol..

[13] Maeda K, Koga T, Akagi J (2017). Interferential current sensory stimulation, through the neck skin, improves airway defense and oral nutrition intake in patients with dysphagia: A double-blind randomized controlled trial. Clin. Interv. Aging..

[14] Nakamori M (2023). Impact of neck percutaneous interferential current sensory stimulation on swallowing function in patients with Parkinson’s disease: A single-arm, open-label study protocol. Contemp. Clin. Trials Commun..

[15] Chan AW (2013). Spirit explanation and elaboration: Guidance for protocols of clinical trials. BMJ.

[16] Postuma RB (2015). MDS clinical diagnostic criteria for Parkinson’s disease. Mov. Disord..

[17] Wakasugi Y (2008). Screening test for silent aspiration at the bedside. Dysphagia.

[18] Sato M, Tohara H, Iida T, Wada S, Inoue M, Ueda K (2012). Simplified cough test for screening silent aspiration. Arch. Phys. Med. Rehabil..

[19] Goetz CG (2008). Movement disorder society-sponsored revision of the Unified Parkinson’s disease rating scale (MDS-UPDRS): Scale presentation and clinimetric testing results. Mov. Disord..

[20] Crary MA, Mann GD, Groher ME (2005). Initial psychometric assessment of a functional oral intake scale for dysphagia in stroke patients. Arch. Phys. Med. Rehabil..

[21] Hiraoka A (2017). Maximum tongue pressure is associated with swallowing dysfunction in ALS patients. Dysphagia.

[22] Kamimura T (2023). Peak expiratory flow, but not tongue pressure, can predict pneumonia development in older adults. Eur. Geriatr. Med..

[23] Jost ST (2023). Levodopa dose equivalency in Parkinson’s disease: Updated systematic review and proposals. Mov. Disord..

[24] Logemann J, Blonsky ER, Boshes B (1973). Lingual control in Parkinson’s disease. Trans. Am. Neurol. Assoc..

[25] Robbins JA, Logemann JA, Kirshner HS (1986). Swallowing and speech production in Parkinson’s disease. Ann. Neurol..

[26] Jolkkonen J, Jenner P, Marsden CD (1995). L-dopa reverses altered gene expression of substance P but not enkephalin in the caudate-putamen of common marmosets treated with MPTP. Brain Res. Mol. Brain Res..

[27] Sasaki H, Sekizawa K, Yanai M, Arai H, Yamaya M, Ohrui T (1997). New strategies for aspiration pneumonia. Intern. Med..

